# UMF-stomata: An unsupervised multi-focus fusion framework for microscopic stomatal phenotyping

**DOI:** 10.1016/j.plaphe.2026.100266

**Published:** 2026-08-15

**Authors:** Xianglong Shi, Aobo Du, Peng Song, Hui Peng, Wanneng Yang, Ruifang Zhai

**Affiliations:** a College of Informatics, Huazhong Agricultural University, Wuhan, Hubei, 430070, China; b College of Plant Science and Technology, Huazhong Agricultural University, Wuhan, Hubei, 430070, China; c National Key Laboratory of Crop Genetic Improvement, Huazhong Agricultural University, Wuhan, Hubei, 430070, China; d Engineering Research Center of Intelligent Technology for Agriculture, Ministry of Education, Huazhong Agricultural University, Wuhan, Hubei, 430070, China; e Hubei Key Laboratory of Agricultural Bioinformatics, Huazhong Agricultural University, Wuhan, Hubei, 430070, China

**Keywords:** Multi-focus image fusion, Unsupervised learning, Stomatal phenotyping, 3D cross-focus modeling, Microscopic image analysis

## Abstract

Stomatal traits are key microscopic phenotypes for evaluating plant physiology, stress responses, and crop breeding potential. However, in vivo high-magnification microscopy often suffers from a shallow depth of field, causing noticeable defocus blur across different spatial locations and making it difficult to capture clear and complete stomatal structures in a single image. Multi-focus image fusion offers a practical solution, yet existing methods typically rely on supervised training, paired data, or hand-crafted rules, limiting their use in real agricultural microscopy scenarios. In this study, we propose an unsupervised multi-focus fusion framework for reconstructing fully focused stomatal microscopic images. The method integrates two-dimensional feature extraction with three-dimensional cross-focal-plane modeling to capture both spatial details and complementary information across focal planes. A max-response-guided spatial gating module is introduced to enhance focused regions while suppressing defocused responses. Additionally, dual sharpness priors based on perceptual features and wavelet high-frequency information enable pixel-wise pseudo-supervised learning without requiring all-in-focus ground-truth images. The model also predicts a probabilistic focal-plane volume for interpretable all-in-focus reconstruction. Experiments on a maize multi-focus image dataset demonstrate that the proposed method achieves superior or competitive performance across multiple fusion metrics, with entropy (EN), edge information preservation (*Q*_AB∕F_), Chen–Blum contrast metric (*Q*_CB_), and visual information fidelity for fusion (VIFF) reaching 7.43, 0.21, 0.41, and 1.01, respectively. Ablation studies confirm the effectiveness of the 3D modeling, spatial gating, and dual-prior sharpness supervision. More importantly, when the fused images serve as input to a YOLO-based stomatal instance segmentation model, the proposed method yields the best segmentation accuracy, with mAP50 and mAP50-95 reaching 0.9937 and 0.9121, respectively. Phenotypic measurements derived from the segmentation masks show high consistency with manual annotations, with the highest coefficient of determination *R*^2^ = 0.97 achieved for stomatal count. These results indicate that the framework can act as an effective front-end module for automated microscopic stomatal phenotyping in agriculture.

## Highlights

• We construct a high-resolution multi-focus stomatal microscopy dataset covering multiple crops, including maize, wheat, cotton, soybean, buckwheat, and rice, providing a multi-crop microscopic multi-focus resource for this research area.
• We propose an end-to-end unsupervised multi-focus stomatal image fusion framework that integrates 2D spatial feature extraction and 3D cross-focal-plane interaction to model complementary focus information across the focal stack. The framework supports an arbitrary number of input focal-plane images and generalizes to unseen crop scenes with different stack depths.
• We design a dual-prior pseudo-supervised focal-plane selection strategy that converts perceptual and wavelet high-frequency sharpness responses into pixel-wise pseudo focus masks to supervise the predicted focal-plane probability volume, enabling interpretable all-in-focus reconstruction without all-in-focus ground-truth images.
• We validate the framework on a downstream instance segmentation task. The proposed fusion images effectively mitigate the impact of defocus blur on feature extraction and significantly improve stomatal detection, instance segmentation, and phenotypic measurement accuracy, providing a reliable automated solution for high-throughput plant micro-phenotyping.

## 1 Introduction

Stomata are key microscopic structures on the leaf epidermis that regulate gas exchange and transpiration between plants and the external environment. Phenotypic traits such as stomatal count, area, perimeter, length, and width are important indicators for crop stress-resistance screening and genetic breeding [[1], [2], [3]]. With the rapid development of high-throughput plant phenomics, fast and accurate extraction of stomatal traits from microscopic images has become an important research topic. In practical in vivo applications, however, microscopic imaging is constrained by optical physics and biological conditions. To capture fine stomatal structures, high-magnification objectives are required, but they inevitably lead to an extremely shallow depth of field [[4], [5], [6]]. In addition, leaf surfaces are usually non-flat and exhibit three-dimensional undulations under in situ observation. As a result, a single exposure cannot provide an all-in-focus image, and the blur becomes more severe as magnification increases. Such partially blurred images, especially from live samples, seriously hinder subsequent automated analysis [7].

In routine stomatal microscopy workflows, extended-depth-of-field techniques have been used to alleviate the shallow depth-of-field problem under high-magnification imaging. For example, Li et al. [7] used the depth-composition function of a Keyence digital microscope to generate clearer stomatal images for subsequent segmentation and measurement. Similarly, Takagi et al. [8] employed the Instant Extended Focal Imaging function implemented in the cellSens platform to facilitate rapid stomatal observation on uneven leaf surfaces. These instrument- or software-based depth-composition workflows are valuable for routine microscopic observation because they can improve image clarity without requiring manual selection of a single focal plane. However, such functions are typically integrated into proprietary commercial imaging platforms, and their core algorithmic details are often difficult to access fully or reproduce independently. Their use commonly depends on dedicated microscopy hardware and licensed software, which may increase image-acquisition costs and limit accessibility and broader adoption across laboratories.

Beyond commercial depth-composition workflows, computational multi-focus image fusion (MFIF) methods provide a more flexible and accessible software-based approach for reconstructing fully focused images from raw multi-focus stacks. However, existing MFIF methods show clear limitations for stomatal phenotyping. Traditional methods can be broadly divided into spatial-domain and transform-domain approaches. Spatial-domain methods usually rely on hand-crafted sharpness measures based on pixel intensity, gradients, or local contrast. Although simple to implement, they are sensitive to noise and lack global consistency [9,10]. Transform-domain methods such as Laplacian pyramids and gradient transforms also depend heavily on hand-crafted features and specific fusion rules, and they often require complex parameter tuning [[11], [12], [13]]. With the rise of deep learning, data-driven fusion methods have gradually become mainstream. Nevertheless, most existing deep fusion methods still depend on fully supervised training with all-in-focus ground truth, which is difficult to obtain in real microscopic agricultural scenarios [14]. Although some studies adopt unsupervised strategies, most of them perform pairwise fusion, which accumulates errors and often produces local blur and artifacts that damage stomatal geometry, while also showing limited generalization in complex microscopic scenes[15]. More importantly, image fusion is fundamentally a pre-processing step for phenotypic analysis [16]. Current automated phenotyping pipelines rely heavily on deep-learning-based segmentation models, whose performance is highly sensitive to image sharpness and structural fidelity. Artifacts and edge blur introduced by poor fusion can severely interfere with feature extraction, causing missed detections, false detections, and inaccurate phenotypic measurements [17,18]. Therefore, a high-quality unsupervised fusion framework is urgently needed to break the visual bottleneck that limits downstream automated phenotyping accuracy.

To address these challenges, we propose an unsupervised fusion-driven stomatal phenotyping framework. First, we develop a multi-focus fusion network that can process an arbitrary number of input images end-to-end. The network combines 2D CNNs and 3D CNNs to model interactions across the focus stack and construct a focus volume, thereby avoiding the error accumulation of iterative pairwise fusion. We further incorporate pretrained perceptual features and frequency-domain priors to guide image generation by measuring the relative importance of feature information, enabling high-quality fusion without all-in-focus ground truth. Second, to validate the practical value of the proposed fusion algorithm, we feed the resulting all-in-focus images into a YOLO instance segmentation model for downstream evaluation. By extracting stomatal geometric traits, we demonstrate that high-quality unsupervised fusion can significantly improve the accuracy of automated stomatal detection, segmentation, and phenotypic measurement.

The main contributions of this work are as follows:

(1) We construct a high-resolution multi-focus stomatal microscopy dataset covering multiple crops, including maize, wheat, cotton, soybean, buckwheat, and rice, providing a multi-crop microscopic multi-focus resource for this research area.
(2) We propose an end-to-end unsupervised multi-focus stomatal image fusion framework that integrates 2D spatial feature extraction and 3D cross-focal-plane interaction to model complementary focus information across the focal stack. The framework supports an arbitrary number of input focal-plane images and generalizes to unseen crop scenes with different stack depths.
(3) We design a dual-prior pseudo-supervised focal-plane selection strategy that converts perceptual and wavelet high-frequency sharpness responses into pixel-wise pseudo focus masks to supervise the predicted focal-plane probability volume, enabling interpretable all-in-focus reconstruction without all-in-focus ground-truth images.
(4) We validate the framework on a downstream instance segmentation task. The proposed fusion images effectively mitigate the impact of defocus blur on feature extraction and significantly improve stomatal detection, instance segmentation, and phenotypic measurement accuracy, providing a reliable automated solution for high-throughput plant micro-phenotyping.

## 2 Materials and methods

### 2.1 Overall pipeline

**Fig. 1:**
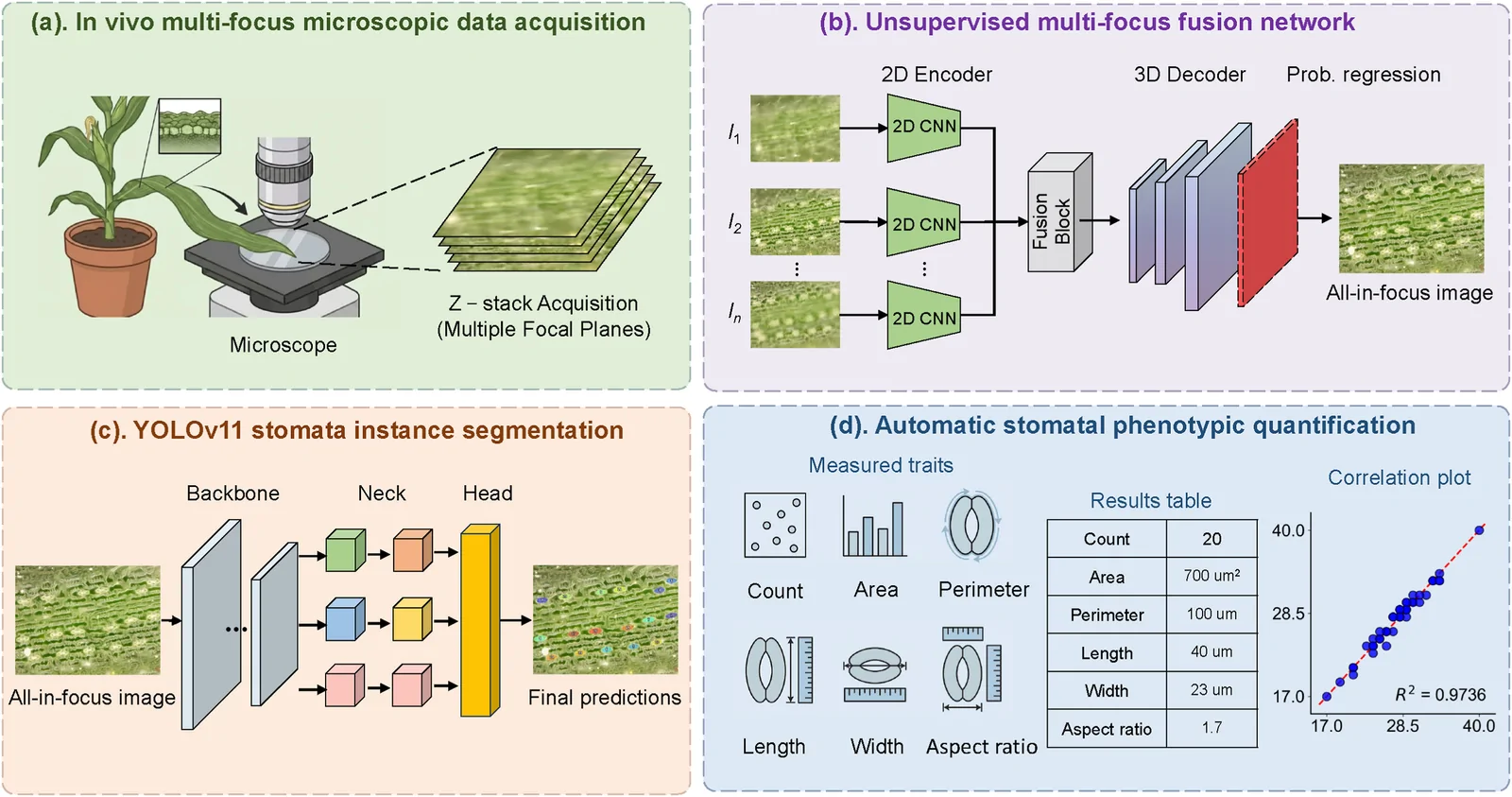
**Pipeline of stomatal phenotyping based on multi-focus fusion.** (a) Acquisition of multi-focal-plane microscopic image stacks; (b) generation of all-in-focus images using the proposed unsupervised multi-focus fusion network; (c) YOLOv11-based stomatal instance segmentation producing pixel-level masks; (d) phenotypic parameter calculation and statistical analysis based on segmentation results.

We establish a complete automated stomatal phenotyping pipeline. As illustrated in Fig. 1, multi-focus microscopic image stacks are first collected from leaf samples. The proposed unsupervised multi-focus fusion network then generates an all-in-focus image. Finally, the fused image is fed into a YOLOv11 instance segmentation model for stomatal detection and pixel-level segmentation. Morphological parameters are computed from the segmentation masks and compared with manual measurements to verify the feasibility and effectiveness of the full pipeline.

### 2.2 Data acquisition and dataset construction

**Fig. 2:**
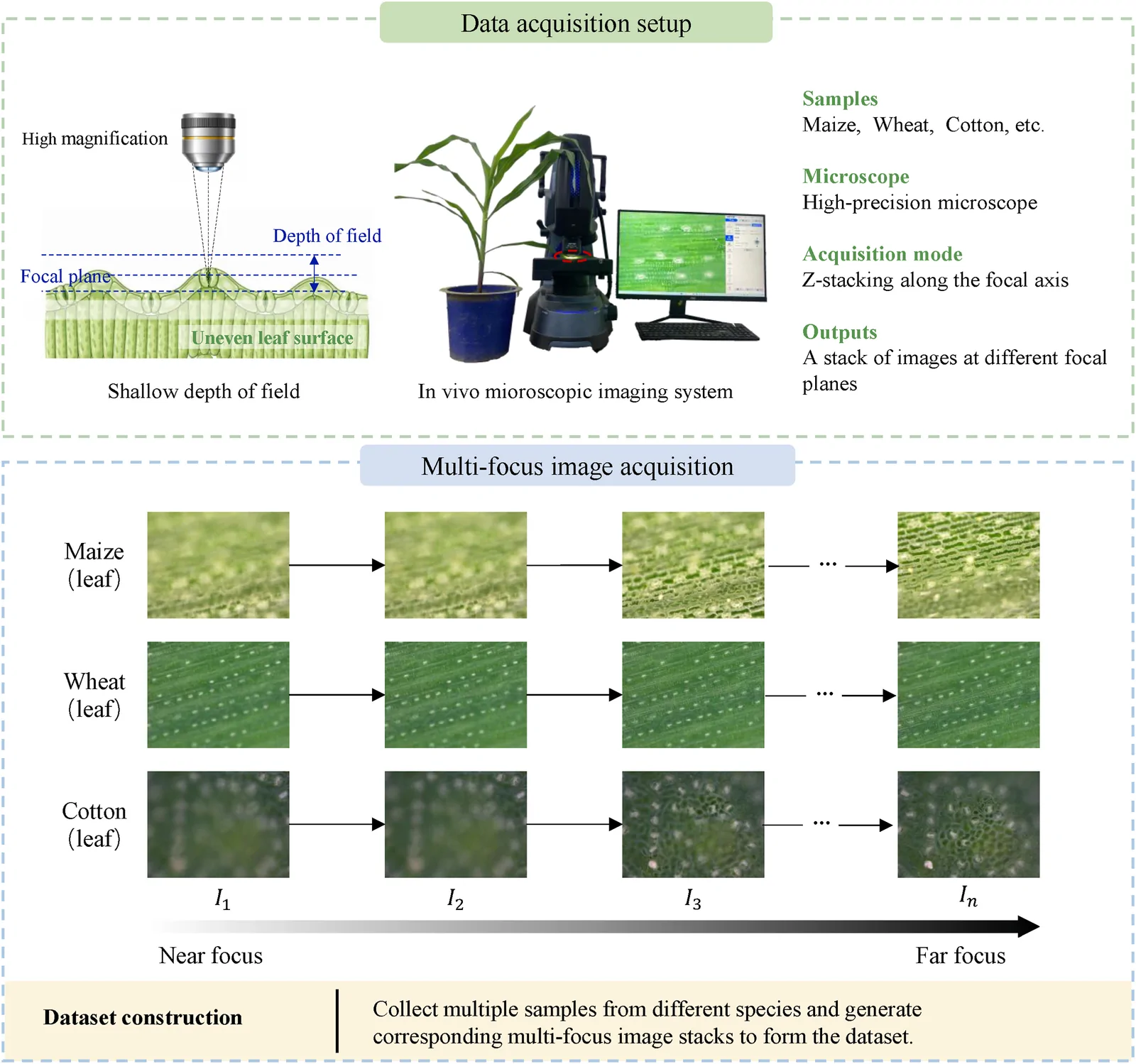
**Overview of multi-focus stomatal microscopy data acquisition and dataset construction.** The workflow illustrates the experimental setup (potted plant samples and high-precision ultra-depth-of-field microscope) and the multi-focus scanning process along the optical axis. Because the depth of field is shallow at high magnification on uneven leaf surfaces, only a limited depth range can be sharply imaged in a single shot, necessitating multi-focus scanning. Representative multi-focus image sequences from maize, wheat, and cotton leaves are shown, ranging from near focus to far focus. These multi-focus image stacks are collected across different species to form a diverse dataset for training, testing, and cross-species generalization.

To build a representative multi-focus stomatal microscopy dataset and evaluate the adaptability and generalization of the proposed method across crops, we collected multi-focus stomatal images from maize and wheat as the main experimental datasets, and additionally collected a smaller number of samples from cotton, soybean, buckwheat, and rice for supplementary generalization tests (Fig. 2). These crops cover both monocotyledonous and dicotyledonous species, increasing data diversity and enabling cross-species robustness analysis. All samples were collected from potted plants during the seedling stage. During sampling, leaves were randomly selected from different leaf positions on each potted plant to capture natural variation in stomatal morphology and density. Before imaging, leaf samples were carefully cleaned to remove dust and impurities while preserving epidermal optical characteristics.

Microscopic imaging was performed using a high-precision ultra-depth-of-field microscope system (Sunny DMS1000, China). To maintain scanning stability, each leaf sample was flattened and fixed on the microscope stage. Considering the micrometer-scale size of stomata, a magnification of 500× was primarily used to balance field of view and boundary-detail resolution. Because depth of field is limited at high magnification and the leaf epidermis is not perfectly flat, a single exposure cannot clearly capture complete stomatal structures. Therefore, a Z-stacking acquisition protocol was adopted. For each field of view, the microscope system automatically performed Z-stack scanning along the optical axis at uniformly spaced focal positions. Images were acquired at a spatial resolution of 2800 × 2100 pixels under LED ring-light illumination. In total, we collected 480 maize stacks, 450 wheat stacks, 50 cotton stacks, 20 soybean stacks, 20 buckwheat stacks, and 10 rice stacks, and each stack contained 21 focal-plane images. Maize, cotton, soybean, buckwheat, and rice were imaged at 500× magnification, whereas wheat was imaged at 200× magnification to provide a larger field of view while preserving sufficient stomatal structural information for fusion and downstream analysis. The image stacks cover the full spatial depth range of the leaf surface and provide rich focus information for fusion. The maize dataset was used for model training, quantitative comparison, and ablation studies, whereas the wheat dataset was used for independent testing and quantitative comparison. The smaller cotton, soybean, buckwheat, and rice datasets were used mainly to evaluate zero-shot generalization to unseen crop scenarios. The basic information of the dataset is shown in Table 1.

**Table 1:** Basic information of the dataset.

Crop	Stacks	Images per stack	Magnification	Acquisition mode
Maize	480	21	500×	Z-stacking
Wheat	450	21	200×	Z-stacking
Cotton	50	21	500×	Z-stacking
Soybean	20	21	500×	Z-stacking
Buckwheat	20	21	500×	Z-stacking
Rice	10	21	500×	Z-stacking

### 2.3 Unsupervised multi-focus image fusion method

#### 2.3.1 Network architecture

**Fig. 3:**
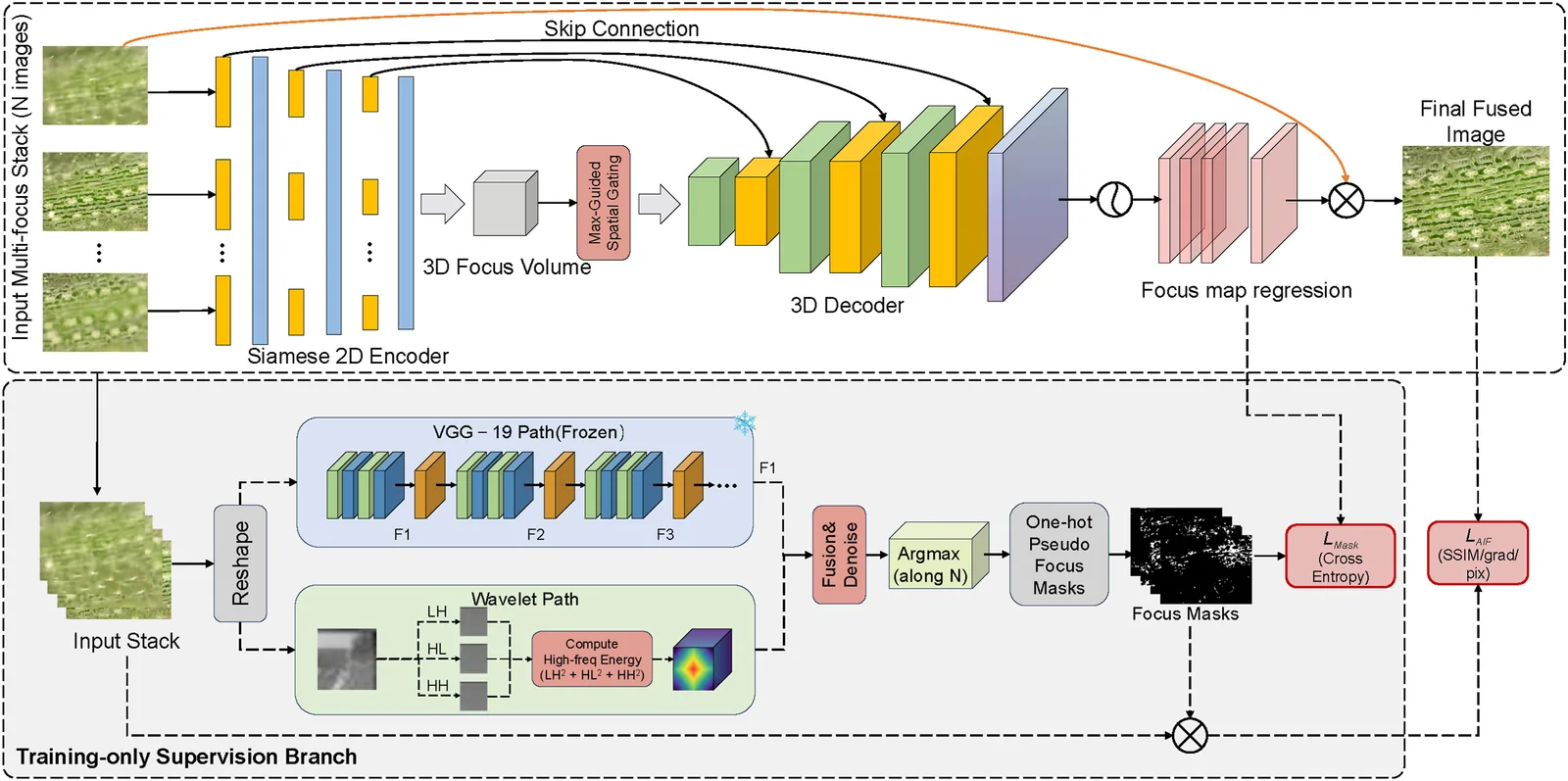
Proposed unsupervised fusion framework.

As shown in Fig. 3, the proposed unsupervised multi-focus fusion network adopts a unified architecture that combines 2D feature extraction with 3D cross-focus modeling. The network first employs a shared 2D encoder to extract local spatial features from each focal plane independently, while introducing cross-focus global guidance. The features are then modeled in the joint spatial-focus domain to construct a focus volume. On this basis, a max-response-guided spatial gating mechanism is introduced to enhance focused regions while suppressing defocused ones. Finally, the resulting features are fed into a 3D decoder to recover spatial resolution, and an interpretable all-in-focus image is reconstructed through a probabilistic focal-plane selection mechanism.

##### 2.3.1.1 2D encoding and cross-focus feature aggregation

Most existing deep learning methods for multi-focus fusion are designed only for two input images. To flexibly handle focus modeling for an arbitrary number of input images, the input multi-focus image stack is first rearranged as

(1) $$X\in R^{B\times N\times C\times H\times W}\to R^{\left(B\cdot N\right)\times C\times H\times W}$$

where *B* denotes the batch size, *N* denotes the number of focal planes in each sample, and *C*, *H*, and *W* denote the number of channels, height, and width of the image, respectively. This rearrangement merges the focal-plane dimension into the batch dimension, allowing each focal-plane image to be treated as an independent sample and directly fed into a shared 2D convolutional encoder for feature extraction.

The feature representation at the *l*-th encoding stage is formulated as

(2) $$F_{n}^{\left(l\right)}=E^{\left(l\right)}\left(X_{n}\right)$$

where $E^{\left(l\right)}$ denotes the *l*-th 2D convolutional block. This design ensures parameter efficiency while enabling the network to effectively capture local sharp structures from different focal planes.

In our implementation, the shared 2D encoder contains four stages with a base width of 16. The four stages produce 16, 32, 64, and 128 output channels, respectively. Each stage consists of two reflection-padded 3 × 3 convolutional layers followed by ReLU activations and a 2 × 2 max-pooling operation. The multi-scale features before pooling are retained as skip connections for the subsequent 3D decoder.

To explicitly model the complementary relationships among focal planes, a cross-focus global max-response guidance mechanism is introduced at each encoding stage. Specifically, for the same spatial location and channel dimension, the maximum response is computed along the focal-plane dimension as:

(3) $$G^{\left(l\right)}\left(x,y,c\right)=\max_{i=1,\ldots ,N}F_{i}^{\left(l\right)}\left(x,y,c\right)$$

This global feature reflects the sharpest and most discriminative response among all focal planes at each spatial position. It is then broadcast and concatenated with the feature map of each focal plane:

(4) $${\tilde{F}}_{n}^{\left(l\right)}=\left[F_{n}^{\left(l\right)},G^{\left(l\right)}\right]$$

By introducing global guidance, the encoder obtains cross-focus sharpness references during local feature extraction, thereby enhancing its ability to respond to the optimal focal regions. After the fourth encoding stage, the pooled local feature and the broadcast stack-wise maximum feature are concatenated, producing a 256-channel representation. A reflection-padded 3 × 3 convolution followed by ReLU activation is then applied to form the bottleneck feature at a spatial resolution of *H*/16 × *W*/16. At the end of the encoder, the features are rearranged into a 3D tensor:

(5) $$H_{3D}\in R^{B\times C\times N\times H\times W}$$

where the third dimension corresponds to the focal-plane index. From this stage onward, the network performs feature modeling in the joint spatial-focus domain, providing a structured representation for subsequent decoding.

##### 2.3.1.2 Max-response-guided spatial gating module

After 2D encoding and cross-focus guidance, the network obtains a focus volume representation that integrates local structural information with global sharpness references. However, feature concatenation alone is still insufficient to fully suppress the interference of defocused regions in the fusion result. To further enhance focused features and suppress redundant blurred information, we design a 3D spatial gating mechanism guided by maximum responses, as illustrated in Fig. S1.

Unlike conventional attention modules that directly generate weights from features themselves, the proposed mechanism first performs max aggregation along the focal-plane dimension:

(6) $$G\left(b,c,h,w\right)=\max_{n}H_{3D}\left(b,c,n,h,w\right)$$

where *G* denotes the strongest response among all focal planes at each spatial location and channel, which can be regarded as the optimal sharpness reference at that position.

The max-response feature is then broadcast along the focal-plane dimension and concatenated with the original 3D feature:

(7) $$Z=\left[H_{3D},\tilde{G}\right]$$

where $\tilde{G}$ denotes the reference feature expanded along the focal-plane dimension. Through this explicit concatenation, the network can directly model the relative relationship between the current focal-plane response and the optimal response.

The concatenated feature is passed through a lightweight gating mapping function composed of two 1 × 1 × 1 3D convolutions and a Sigmoid activation, and the original 3D feature is modulated element-wise as:

(8) $$\begin{array}{ll} M & \;=\sigma \left(f\left(Z\right)\right),\\ H_{3D}^{\prime } & \;=H_{3D}⊙M \end{array}$$

Here, **Z** denotes the concatenated 3D feature, *f*(⋅) denotes the nonlinear mapping implemented by two 1 × 1 × 1 3D convolutions, and *σ*(⋅) denotes the Sigmoid activation function. **M** ∈ [0, 1] is the gating weight tensor, which has the same size as the original 3D feature **H**_3*D*_ and is used to characterize the relative importance of each spatial location and focal plane. ⊙ denotes element-wise multiplication, through which the original features are adaptively reweighted.

In essence, this design constructs an explicit cross-focus comparison mechanism, enabling the network to adaptively enhance features that are close to the optimal response at each spatial location while suppressing defocused responses that deviate from the optimal focal plane. Compared with complex self-attention structures, the proposed method does not require computation of high-order similarity matrices, leading to lower computational cost, easier optimization, and clearer physical interpretability.

##### 2.3.1.3 3D decoding and fusion reconstruction

After processing by the max-response-guided spatial gating module, the network obtains a discriminative 3D feature representation in the joint spatial-focus domain. The decoder consists of four 3D decoding blocks with channel configurations of 256 → 128, 128 → 64, 64 → 32, and 32 → 16. At each stage, trilinear interpolation is applied to align the upsampled feature with the corresponding encoder skip feature in spatial resolution while keeping the focal-plane dimension consistent. The upsampled feature is then processed by a reflection-padded 3 × 3 × 3 convolution, BatchNorm3D, and ReLU activation. It is concatenated with the corresponding 3D skip feature and further refined by a second reflection-padded 3 × 3 × 3 convolution followed by BatchNorm3D and ReLU activation:

(9) $$H^{\left(l\right)}=D_{2}^{\left(l\right)}\left(\left[D_{1}^{\left(l\right)}\left({Up}_{3D}\left(H^{\left(l+1\right)}\right)\right),S^{\left(l\right)}\right]\right),$$

where $D_{1}^{\left(l\right)}$ and $D_{2}^{\left(l\right)}$ denote the first and second 3D convolutional refinement blocks at the *l*-th decoding stage, respectively; Up_3*D*_(⋅) denotes trilinear interpolation, and **S**^(*l*)^ denotes the corresponding 3D skip feature from the encoder. This decoding strategy progressively restores spatial resolution while propagating multi-scale spatial details and cross-focal-plane information.

Finally, two reflection-padded 3 × 3 × 3 convolutions with channel configuration 16 → 16 → 1 generate a one-channel focal-plane cost volume. A softmax operation is applied along the focal-plane dimension to obtain normalized fusion weights, and the final all-in-focus image is reconstructed by the weighted summation of the input focal-plane images:

(10) $$C\in R^{B\times N\times 1\times H\times W}$$

(11) $$M_{i}\left(x,y\right)=\frac{\exp\left(C_{i}\left(x,y\right)\right)}{\sum_{k=1}^{N}\exp\left(C_{k}\left(x,y\right)\right)}$$

(12) $$Y\left(x,y\right)=\overset{N}{\underset{i=1}{\sum }}M_{i}\left(x,y\right)X_{i}\left(x,y\right)$$

This fusion strategy can be regarded as a soft focal-plane selection process for each pixel. Unlike conventional methods that rely on hard thresholding or rule-based focal-plane switching, the proposed method continuously models the contribution of different focal planes at the pixel level, thereby effectively avoiding discontinuities near focus boundaries.

### 2.4 Unsupervised sharpness modeling

In multi-focus image fusion, the sharpness of different focal planes varies significantly across spatial locations. To characterize such differences under an unsupervised setting, we perform unsupervised sharpness modeling of the input images from two complementary perspectives, namely perceptual features and high-frequency structural information, thereby providing reliable pseudo-supervision for network training.

Given an input multi-focus image stack

(13) $$I={\left\{I_{n}\right\}}_{n=1}^{N},\;I_{n}\in R^{C\times H\times W}$$

where *N* denotes the number of focal planes and *I*_*n*_ denotes the image corresponding to the *n*-th focal plane.

First, shallow perceptual features are extracted using a pretrained and frozen VGG network. To ensure feature stability, the input image is normalized before being fed into the network, and the resulting feature maps are aggregated along the channel dimension to obtain a pixel-wise perceptual response map:

(14) $$R_{n}^{\text{VGG}}\left(x\right)=\underset{c}{\sum }\phi_{c}\left(I_{n}\left(x\right)\right)$$

where *ϕ*(⋅) denotes the convolutional feature mapping of the VGG network, and *x* denotes the spatial position.

Meanwhile, to enhance sensitivity to local structures and edge information, a discrete wavelet transform (DWT) is applied to the input image, and the energy of the high-frequency subbands is used to characterize detail intensity:

(15) $$R_{n}^{\text{DWT}}\left(x\right)=\underset{b\in \left\{LH,HL,HH\right\}}{\sum }\|W_{b}\left(I_{n}\left(x\right)\right){\|}_{2}^{2}$$

where *W*_*b*_(⋅) denotes the corresponding wavelet high-frequency subband.

The two types of responses are fused in the low-resolution space to obtain the initial sharpness response map:

(16) $$R_{n}\left(x\right)=R_{n}^{\text{VGG}}\left(x\right)+R_{n}^{\text{DWT}}\left(x\right)$$

In Eq. (16), the VGG-based response and the DWT high-frequency energy are used as complementary focus cues. The shallow VGG features capture local texture and edge saliency in a learned feature space, whereas the DWT high-frequency energy emphasizes fine structural details and sharp transitions that are typically stronger in in-focus regions. We therefore combine the two terms to construct a more robust pseudo-focus response map. Equal weighting is adopted as a simple and stable setting for unsupervised pseudo-label generation, avoiding additional trainable or manually tuned weighting parameters. To suppress isolated noisy responses and improve spatial consistency, the fused response map is subsequently smoothed using local averaging. Based on the resulting sharpness responses, a maximum-response selection is performed along the focal-plane dimension to assign the sharpest focal plane to each spatial position:

(17) $$M\left(x\right)=\arg\max_{n}R_{n}\left(x\right)$$

A one-hot spatial mask is then constructed as

(18) $$M\in {\left\{0,1\right\}}^{N\times H\times W}$$

where **M**_*n*_(*x*) = 1 indicates that the *n*-th focal plane is considered the sharpest at position *x*. This mask is generated entirely in an input-adaptive manner without any manual annotation, and it serves as a pixel-level pseudo-supervisory signal during training.

### 2.5 Loss function

In unsupervised multi-focus image fusion, the absence of all-in-focus reference images requires the training process to rely on effective constraints constructed from the input data itself. Based on the sharpness pseudo-supervision mask constructed in Section 2.4, we design the optimization objective from two aspects, namely reconstruction consistency and focal-plane discriminability, so as to guide the network toward pixel-wise optimal focal-plane selection and high-quality image reconstruction.

Specifically, the fused result is expanded along the focal-plane dimension and aligned with the input images to construct an unsupervised reconstruction loss. The pixel reconstruction loss is defined as

(19) $$L_{\mathrm{pix}}=\frac{1}{N}\overset{N}{\underset{i=1}{\sum }}\|M_{i}⊙{\hat{I}}_{i}-M_{i}⊙I_{i}{\|}_{2}^{2}$$

where $M_{n}⊙{\hat{I}}_{n}$ and **M**_*n*_ ⊙ *I*_*n*_ denote the fused result and the input stack under mask constraint, respectively, and ⊙ denotes element-wise multiplication. This loss ensures that the fused result is as close as possible to the corresponding source image in the regions identified as sharp.

To further enhance structural consistency between the fused result and the sharp regions, a structural similarity loss is introduced:

(20) $$L_{\mathrm{ssim}}=1-SSIM\left(M_{i}⊙{\hat{I}}_{i},M_{i}⊙I_{i}\right)$$

Meanwhile, to better preserve edges and high-frequency details, a gradient consistency loss is introduced:

(21) $$L_{\mathrm{grad}}=\frac{1}{N}\overset{N}{\underset{i=1}{\sum }}\|\nabla \left(M_{i}⊙{\hat{I}}_{i}\right)-\nabla \left(M_{i}⊙I_{i}\right){\|}_{2}^{2}$$

where ∇(⋅) denotes the image gradient operator. This term effectively constrains edge continuity and texture preservation in the sharp regions of the fused result.

By combining the above three terms, the fusion reconstruction loss is obtained as

(22) $$L_{\mathrm{AIF}}=L_{\mathrm{pix}}+L_{\mathrm{ssim}}+L_{\mathrm{grad}}$$

Here, the pixel consistency loss, structural similarity loss (SSIM), and gradient loss constrain brightness fidelity, structural consistency, and edge continuity, respectively.

In addition to constraining the final fused image, we further supervise the focal-plane probability volume predicted by the network to improve its ability to identify pixel-wise sharp focal planes. Specifically, the pseudo-supervision mask generated from the sharpness response is regarded as a pixel-level target distribution, and a distribution cross-entropy constraint is imposed on the predicted probability volume:

(23) $$L_{\text{Mask}}=-\overset{N}{\underset{i=1}{\sum }}\underset{x,y}{\sum }M_{i}\left(x,y\right)\log P_{i}\left(x,y\right)$$

where *P*_*n*_(*x*, *y*) denotes the probability predicted by the network for the *n*-th focal plane at position (*x*, *y*), and **M**_*n*_(*x*, *y*) denotes the corresponding pseudo-supervised distribution. This loss encourages the network to output focal-plane selection results consistent with the pseudo-supervision, thereby learning pixel-wise sharp-region discriminability.

Finally, the overall optimization objective is defined as

(24) $$L=L_{\mathrm{AIF}}+L_{\mathrm{Mask}}$$

Through the above design, the network can be stably trained using only the multi-focus input itself, without any all-in-focus reference image or explicit depth annotation, while adaptively enhancing sharp structures and suppressing defocused regions.

## 3 Experiments and results

### 3.1 Implementation details

For fusion training, we used the self-collected maize multi-focus microscopy dataset. A total of 480 maize stacks were selected, each containing 21 focal-plane images. The data were split into training, validation, and test subsets at a ratio of 8:1:1. To increase the number of training samples and improve local-structure modeling, random crops of size 128 × 128 were extracted from the training stacks, resulting in 9600 multi-focus stacks for training. To evaluate cross-crop generalization, the multi-focus datasets of wheat, cotton, buckwheat, soybean and rice were used only during testing and were not involved in training or parameter tuning. The fusion network was trained end-to-end on an NVIDIA GeForce RTX 3090 GPU using the Adam optimizer with a batch size of 32, an initial learning rate of 1 × 10^−4^, and 50 epochs.

For downstream stomatal instance segmentation, a pretrained YOLO11x-seg model was trained on the manually annotated ultra-depth-of-field stomatal image dataset. The dataset was randomly divided at the field-of-view level into training, validation, and test sets at a ratio of 8:1:1. Training was performed for 200 epochs with a batch size of 4 using the SGD optimizer on an NVIDIA GeForce RTX 3090 GPU.

### 3.2 Evaluation metrics

To comprehensively evaluate the proposed method in both multi-focus image fusion and downstream stomatal phenotyping, we establish a three-level evaluation framework, including fusion quality, segmentation accuracy, and phenotypic measurement consistency. Specifically, objective fusion metrics are used to assess image quality, instance segmentation metrics are adopted to evaluate downstream detection and segmentation performance, and regression error and correlation metrics are employed to quantify the agreement between automatically extracted phenotypes and manual measurements, thereby validating the effectiveness and reliability of the proposed method in agricultural phenotyping tasks.

#### 3.2.1 Evaluation metrics for multi-focus image fusion

To quantitatively evaluate the performance of multi-focus image fusion in terms of information preservation, edge structure retention, and consistency with human visual perception, five widely used objective metrics are adopted: entropy (EN), mutual information (MI), edge-based similarity (*Q*_*AB*/*F*_), the Chen–Blum visual contrast metric (*Q*_*CB*_), and visual information fidelity for fusion (VIFF).

Assume that the input multi-focus image sequence consists of *N* images, denoted as {*I*_1_, *I*_2_, *…*, *I*_*N*_}, and the fused all-in-focus image is denoted as *F*.

(1) Entropy (EN)

Entropy measures the total amount of information contained in the fused image. A higher EN value indicates richer details and greater information content. It is defined as:

(25) $$EN=-\overset{L-1}{\underset{l=0}{\sum }}p_{l}\log_{2}p_{l}$$

where *L* is the number of gray levels, and *p*_*l*_ represents the probability of gray level *l* in the fused image *F*.

(2) Mutual Information (MI)

Mutual information quantifies the amount of information that the fused image inherits from the source images from an information-theoretic perspective. A higher MI value indicates stronger correlation between the fused image and the source images. For multi-source inputs, the total mutual information is defined as:

(26) $$MI=\overset{N}{\underset{i=1}{\sum }}I\left(I_{i},F\right)$$

where

(27) $$I\left(I_{i},F\right)=\sum p_{I_{i},F}\left(x,y\right)\log_{2}\frac{p_{I_{i},F}\left(x,y\right)}{p_{I_{i}}\left(x\right)p_{F}\left(y\right)}$$

Here, $p_{I_{i},F}$ is the joint probability distribution, and $p_{I_{i}}$ and *p*_*F*_ are the marginal probability distributions.

(3) Edge-Based Similarity (*Q*_*AB*/*F*_)

This metric evaluates how well the fused image preserves edge and gradient information from the source images. Values closer to 1 indicate better edge preservation. Its generalized form for multi-focus inputs is:

(28) $$Q_{AB/F}=\frac{\sum_{i=1}^{N}\sum_{x,y}Q_{I_{i},F}\left(x,y\right)\;w_{I_{i}}\left(x,y\right)}{\sum_{i=1}^{N}\sum_{x,y}w_{I_{i}}\left(x,y\right)}$$

where $Q_{I_{i},F}\left(x,y\right)$ represents the edge strength and orientation preservation between the source image *I*_*i*_ and the fused image *F*, and $w_{I_{i}}\left(x,y\right)$ is the local gradient weight emphasizing salient edge regions.

(4) Chen–Blum Metric (*Q*_*CB*_)

The *Q*_*CB*_ metric incorporates models of the human visual system (HVS), such as contrast sensitivity and masking effects, to evaluate fusion quality in a perceptually meaningful way. A higher value indicates more natural visual contrast. It is defined as:

(29) $$Q_{CB}=\frac{\sum_{i=1}^{N}\sum_{x,y}Q_{I_{i},F}\left(x,y\right)\;\lambda_{I_{i}}\left(x,y\right)}{\sum_{i=1}^{N}\sum_{x,y}\lambda_{I_{i}}\left(x,y\right)}$$

where $Q_{I_{i},F}\left(x,y\right)$ denotes the perceptual contrast quality after HVS filtering, and $\lambda_{I_{i}}\left(x,y\right)$ is the saliency weight.

(5) Visual Information Fidelity for Fusion (VIFF)

VIFF evaluates the visual information distortion between source and fused images based on natural scene statistics (NSS) and HVS models across multiple scales. A higher VIFF value indicates less distortion and higher visual fidelity. It is defined as:

(30) $$VIFF=\overset{K}{\underset{k=1}{\sum }}p_{k}\overset{N}{\underset{i=1}{\sum }}\frac{I\left(C,F_{k,i}\right)}{I\left(C,E_{k}\right)}$$

where *k* denotes the scale level, *p*_*k*_ is the corresponding weight, and *I*(⋅, ⋅) measures the mutual information between the reference and fused images.

#### 3.2.2 Evaluation metrics for image segmentation

To quantitatively evaluate the impact of the proposed fusion method on downstream automated phenotyping tasks, standard metrics from instance segmentation are employed to assess the outputs of the YOLO model, including Precision, Recall, and mean Average Precision (mAP).

First, the Intersection over Union (IoU) between the predicted mask *M*_pred_ and the ground-truth mask *M*_gt_ is computed as:

(31) $$IoU=\frac{\left|M_{\text{pred}}\cap M_{\text{gt}}\right|}{\left|M_{\text{pred}}\cup M_{\text{gt}}\right|}$$

Based on a predefined IoU threshold, true positives (TP), false positives (FP), and false negatives (FN) are determined. Precision and Recall are defined as:

(32) $$\begin{array}{ll} Precision & =\frac{TP}{TP+FP},\\ Recall & =\frac{TP}{TP+FN} \end{array}$$

To comprehensively evaluate model performance, Average Precision (AP) is calculated as the area under the precision–recall (P–R) curve. Following the COCO evaluation protocol, two key metrics are used:

mAP50 (mAP@0.5): Average precision at a fixed IoU threshold of 0.5, reflecting basic object localization and segmentation capability.

mAP50–95 (mAP@0.5:0.95): The mean AP over IoU thresholds ranging from 0.5 to 0.95 with a step size of 0.05:

(33) $$mAP_{50-95}=\frac{1}{10}\overset{0.95}{\underset{t=0.5}{\sum }}AP_{\mathrm{IoU=t}}$$

This metric imposes stricter requirements on mask boundary accuracy. It not only requires correct object detection but also highly precise pixel-level segmentation. Therefore, it effectively reflects the contribution of high-quality fused images in reducing focus artifacts and improving boundary reconstruction for downstream tasks.

#### 3.2.3 Evaluation metrics for phenotypic trait extraction

To quantitatively evaluate the accuracy of stomatal phenotypic traits derived from segmentation results, the automatically extracted measurements are compared with manually annotated ground-truth values. Let *y*_*i*_ denote the ground-truth phenotypic value and ${\hat{y}}_{i}$ denote the corresponding predicted value, where *i* = 1, *…*, *N* indexes the test samples.

First, the Mean Absolute Error (MAE) is used to measure the average absolute deviation between predicted and ground-truth values:

(34) $$MAE=\frac{1}{N}\overset{N}{\underset{i=1}{\sum }}|{\hat{y}}_{i}-y_{i}|$$

This metric is less sensitive to outliers and provides an intuitive measure of the average prediction error.

Second, the Root Mean Square Error (RMSE) is introduced to capture the overall magnitude of prediction errors:

(35) $$RMSE=\sqrt{\frac{1}{N}\overset{N}{\underset{i=1}{\sum }}{\left({\hat{y}}_{i}-y_{i}\right)}^{2}}$$

Compared to MAE, RMSE penalizes larger errors more heavily and is therefore more sensitive to extreme deviations.

To further evaluate the linear consistency between predicted and ground-truth phenotypic values, the coefficient of determination (*R*^2^) is computed as:

(36) $$R^{2}=1-\frac{\sum_{i=1}^{N}{\left({\hat{y}}_{i}-y_{i}\right)}^{2}}{\sum_{i=1}^{N}{\left(y_{i}-\bar{y}\right)}^{2}}$$

where $\bar{y}$ represents the mean of the ground-truth values. A higher *R*^2^ indicates better agreement between predicted and true values.

These evaluation metrics are applied to multiple stomatal phenotypic traits, including stomatal count, length, width, area, perimeter and aspect ratio, thereby providing a comprehensive assessment of the accuracy and reliability of the proposed method in microscopic stomatal phenotyping analysis.

### 3.3 Fusion performance evaluation

To comprehensively evaluate the effectiveness and robustness of the proposed fusion network in microscopic scenarios, we compare it with representative traditional and deep-learning-based methods. The traditional methods include CVT [19], DSIFT [20], and NSCT [21]. The deep-learning baselines include U2Fusion [22], SDNet [23], MFF-GAN [24], and SwinFusion [25]. For deep-learning comparison methods, we used publicly available code and pretrained weights and evaluated them under a unified inference protocol. Specifically, all input images were normalized according to the requirements of the original implementation of each method. For methods that only support two-image input, the multi-focus stack was fused through a fixed pairwise iterative strategy following the original focal-plane acquisition order. No additional image enhancement or post-processing beyond that required by the original implementations was applied. Detailed settings are provided in Supplementary Table S1.

#### 3.3.1 Qualitative evaluation

**Fig. 4:**
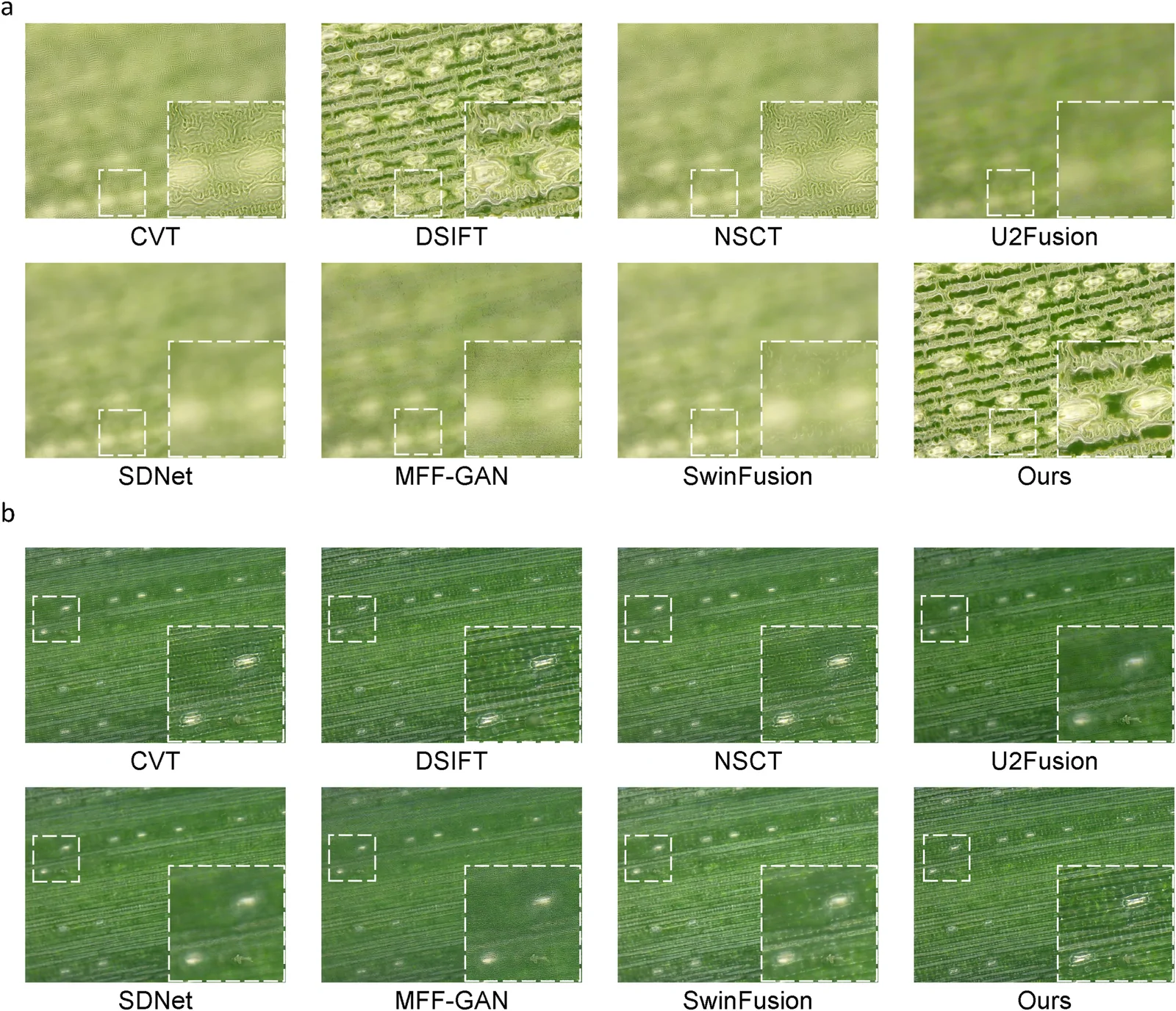
Qualitative comparison of multi-focus image fusion methods on the maize and wheat stomatal datasets, where panel (a) shows maize and panel (b) shows wheat.

Fig. 4(a) and (b) show the fusion results of different methods on the maize and wheat datasets, respectively. The visual differences between methods are clearly data-dependent. In the wheat dataset, where depth variation is relatively mild, most traditional methods can produce acceptable fusion results. Although some methods suffer from information loss and slight local blur, the overall structures remain reasonably clear. In the maize dataset, however, where depth variation is more complex and defocus blur is more severe, the limitations of traditional methods become much more obvious. Only DSIFT maintains global structural clarity to some extent, whereas the multiscale-transform-based NSCT and CVT introduce obvious blur and ringing artifacts in high-frequency regions, severely damaging the continuity of stomatal boundaries.

Most deep-learning methods also face major challenges on both datasets. U2Fusion, SDNet, SwinFusion, and MFF-GAN generally produce globally blurred outputs and fail to integrate sharp information from different focal planes effectively. In addition to blur, MFF-GAN introduces obvious noise in high-frequency regions, further reducing structural fidelity. These degradations are mainly caused by error accumulation during fusion and indicate that existing methods still struggle to preserve both structure and fine details in complex microscopic multi-focus scenarios.

By contrast, the proposed method shows stronger stability and adaptability on both datasets. It explicitly models structural consistency across focal planes and achieves smooth and natural focus transitions under complex defocus conditions. The fused images retain clear textures and continuous structures in different focal regions, with complete stomatal contours, sharp boundaries, and no obvious artifacts or over-smoothing. To further analyze the transfer of source-image information into fused outputs, we perform residual analysis by visualizing pixel-wise differences between the fused image and a reference source image containing the largest clear region. Ideally, the residual map should be close to zero except for a small amount of random noise. As shown in Fig. S2, most methods still exhibit obvious structural residuals around focal-plane boundaries, indicating inaccurate identification of the optimal focal region or information loss during fusion. In contrast, the residual map of our method contains only sparse responses, with almost no obvious residuals around boundaries, demonstrating more accurate integration of multi-focus information and more effective pixel-wise focal-plane selection.

To further explain the fusion-selection mechanism, Fig. S3 shows the input multi-focus stack together with the corresponding decision maps. The high-response regions in the decision maps align well with clear regions in the source images, indicating that the model adaptively selects better focal-plane information according to spatial sharpness differences. This result shows that the probabilistic focal-plane selection mechanism is interpretable and provides strong support for high-quality fusion.

#### 3.3.2 Quantitative evaluation

To objectively and systematically evaluate the fusion performance of different methods, we conduct quantitative analysis using the metrics described in Section 3.2.1, including entropy (EN), edge information preservation (*Q*_*AB*/*F*_), mutual information (MI), Chen–Blum contrast metric (*Q*_*CB*_), and visual information fidelity for fusion (VIFF). Table 2 report the statistical results on 48 maize stacks and 45 wheat stacks, respectively, where the best and second-best results are highlighted in red and underlined.

**Table 2:** Quantitative evaluation results on maize and wheat stomatal datasets (Best results are highlighted in red, and the second-best results are underlined).

Method	Maize					Wheat				
	EN *↑*	MI *↑*	*Q*_AB∕F_*↑*	*Q*_CB_*↑*	VIFF *↑*	EN *↑*	MI *↑*	*Q*_AB∕F_*↑*	*Q*_CB_*↑*	VIFF *↑*
CVT	6.71	4.29	0.16	0.32	0.29	6.50	5.80	0.20	0.43	0.53
DSIFT	7.32	6.35	0.19	0.43	0.88	6.54	8.87	0.23	0.51	0.92
NSCT	6.69	4.49	0.17	0.33	0.23	6.45	6.23	0.22	0.43	0.51
MFF-GAN	7.12	2.97	0.08	0.13	0.17	7.45	1.06	0.05	0.22	0.17
SDNet	6.17	10.97	0.13	0.05	0.01	6.04	7.75	0.12	0.16	0.23
SwinFusion	6.06	12.63	0.12	0.13	0.09	6.14	14.43	0.15	0.36	0.50
U2Fusion	5.87	10.30	0.13	0.11	0.08	6.07	9.14	0.13	0.30	0.28
Ours	7.43	9.26	0.21	0.41	1.01	6.52	10.10	0.25	0.53	0.92

Overall, the proposed method achieves the best or second-best performance across most metrics, demonstrating stable and superior overall performance. In particular, our method obtains the best results on both *Q*_*AB*/*F*_ and VIFF across the two datasets, indicating that the fused images are highly consistent with the original focused information in terms of structural fidelity and visual perception.

For the *Q*_*CB*_ metric, our method ranks second on the maize dataset, further confirming its effectiveness in preserving contrast and visual naturalness. The MI metric reflects the ability of the fused image to integrate information from multiple source images. Although some deep learning-based methods achieve higher MI values, visual inspection reveals that these gains are often due to the introduction of noise and redundant information rather than meaningful detail enhancement, suggesting that a single metric cannot fully characterize fusion quality.

**Fig. 5:**
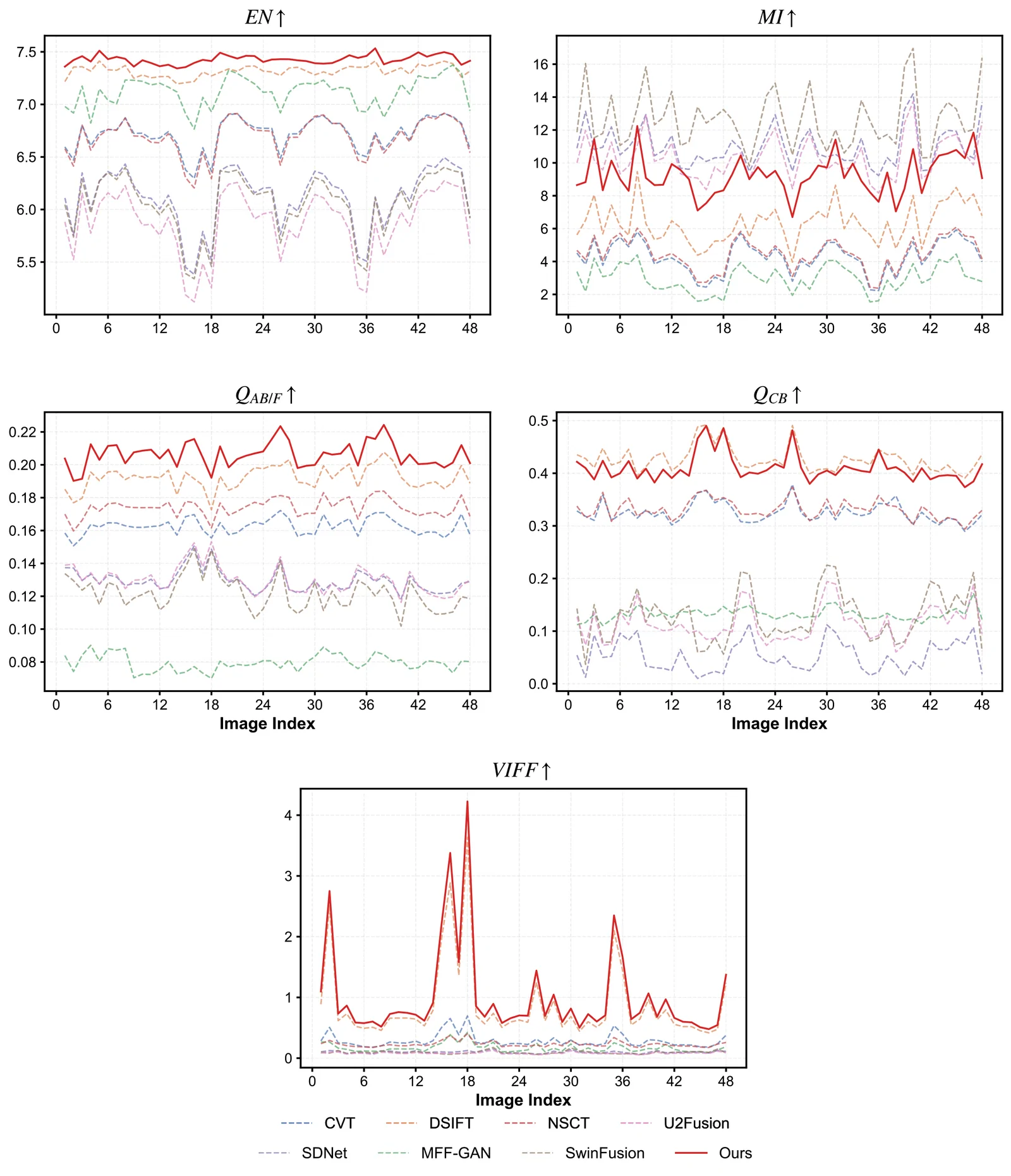
Quantitative comparison of fusion performance across all maize test samples. The x-axis represents individual image stacks, and the y-axis denotes evaluation metric values. Arrows indicate the optimization direction of each metric ( *↑*: higher is better).

In addition, Fig. 5 presents detailed per-sample quantitative results on the maize dataset, providing a more comprehensive view of the stability and robustness of our method across diverse test cases.

In summary, the proposed method achieves optimal or near-optimal performance in terms of information preservation, structural reconstruction, and perceptual consistency, demonstrating superior overall performance and enabling stable, high-quality fusion in complex microscopic multi-focus scenarios.

### 3.4 Ablation study

To systematically evaluate the contributions of key components in the proposed multi-focus fusion framework, ablation experiments were conducted on the maize dataset. Specifically, different modules were selectively removed or combined, including the max-response-guided spatial gating module (MGSG), three-dimensional convolutional modeling (3DConv), perceptual sharpness modeling based on VGG features (VGG Prior), and high-frequency sharpness modeling based on discrete wavelet transform (DWT Prior). All variants were evaluated under the same training and testing settings, and the quantitative results are reported in Table 3.

**Table 3:** Ablation study on the maize stomatal dataset.

MGSG	3DConv	VGG Prior	DWT Prior	EN *↑*	MI *↑*	*Q*_AB∕F_*↑*	*Q*_CB_*↑*	VIFF *↑*
	*✓*	*✓*	*✓*	7.40	8.86	0.21	0.41	0.97
*✓*		*✓*	*✓*	7.36	8.49	0.21	0.41	0.94
*✓*	*✓*		*✓*	7.42	8.91	0.21	0.41	1.00
*✓*	*✓*	*✓*		7.26	7.74	0.21	0.41	0.84
*✓*	*✓*	*✓*	*✓*	7.43	9.26	0.21	0.41	1.01

Overall, each module contributes to the fusion performance to varying degrees. When only 3DConv and sharpness modeling are retained (without MGSG), the model achieves reasonable performance on EN and MI, but the VIFF score is 0.97, indicating that although the information content is relatively high, the visual fidelity can still be improved. This suggests that relying solely on 3D feature modeling is insufficient to effectively suppress defocused information. When MGSG is introduced while removing 3DConv, the performance degrades (MI drops from 8.86 to 8.49, and VIFF from 0.97 to 0.94), indicating that although MGSG enhances focused regions, its effectiveness is limited without 3D contextual modeling. This demonstrates a clear synergy between MGSG and 3D feature modeling.

Further analysis of different sharpness modeling strategies shows that removing the DWT Prior while retaining the VGG Prior leads to a VIFF score of 1.00, reflecting strong visual fidelity. In contrast, removing the VGG Prior while retaining only the DWT Prior significantly degrades performance (MI decreases to 7.74 and VIFF to 0.84). This indicates that although high-frequency information can enhance local details, it may introduce noise or unstable responses when used alone, whereas perceptual features provide more stable global structural modeling. When both VGG Prior and DWT Prior are incorporated, the model achieves better performance across all metrics, demonstrating their complementary roles: the former captures semantic and structural information, while the latter enhances edges and fine details.

Finally, when all modules (MGSG + 3DConv + VGG Prior + DWT Prior) are included, the model achieves the best performance. This result indicates that the modules work synergistically within the overall framework: 3DConv enables cross-focal-plane modeling, MGSG performs selective enhancement of focused regions, and the dual priors jointly provide stable unsupervised sharpness constraints.

### 3.5 Downstream segmentation and stomatal phenotyping

To validate the practical value of the proposed fusion method for high-throughput phenotyping, we first trained a YOLO instance segmentation model in a fully supervised manner using high-quality all-in-focus maize images synthesized by the ultra-depth-of-field microscope and strictly annotated by experts. During testing, the all-in-focus images generated by different fusion algorithms from the original multi-focus stacks were sequentially fed into the pretrained YOLO model for inference. Segmentation accuracy and phenotypic parameter extraction error were then jointly evaluated.

#### 3.5.1 Instance segmentation performance

**Fig. 6:**
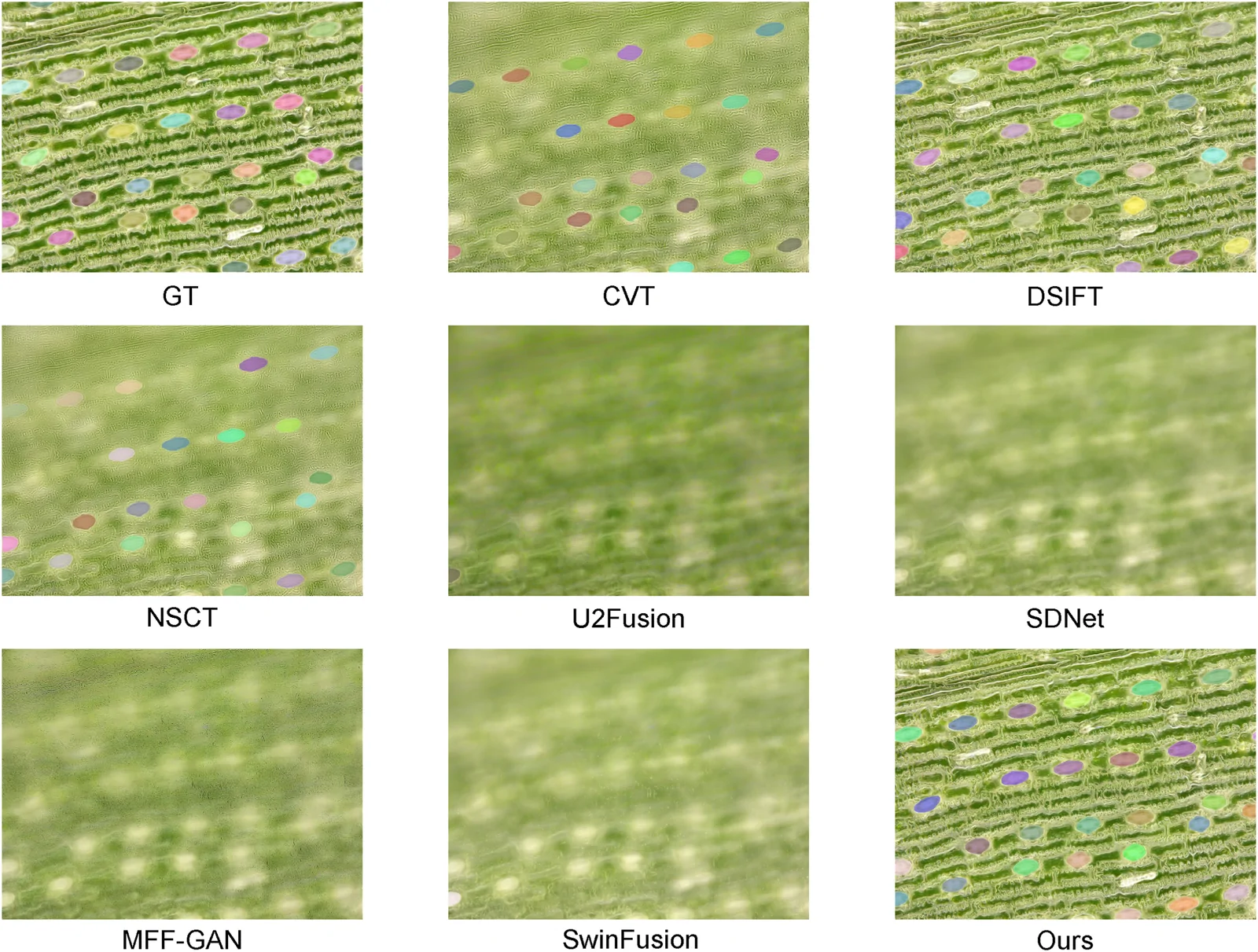
Visual comparison of stomatal instance segmentation results obtained using different fusion methods.

As shown in Table 4 and Fig. S4, when directly using partially focused raw images or low-quality fusion results, especially those from traditional methods with ringing artifacts, the YOLO model suffers clear decreases in recall and mAP50-95, indicating that blur and artifacts seriously interfere with precise localization of tiny stomatal boundaries. In contrast, when using the fused images generated by our method, the YOLO model achieves the best segmentation performance. In particular, on the highly demanding mAP50-95 metric, our method improves by about 4% over the second-best fusion algorithm. Furthermore, as shown in Fig. 6, visual comparisons reveal that our method produces more complete boundaries and better instance separation, while competing methods suffer from missed detections and boundary fragmentation. This strongly demonstrates that the proposed fusion method effectively eliminates defocus blur and fusion artifacts and provides highly clear and faithful semantic features for the downstream segmentation model.

**Table 4:** Quantitative instance segmentation results using images fused by different methods.

Method	mAP50 *↑*	mAP50-95 *↑*	Precision *↑*	Recall *↑*
CVT	0.9764	0.7629	0.9932	0.9376
DSIFT	0.9847	0.8749	0.9768	0.9529
NSCT	0.9790	0.7719	0.9900	0.9442
MFF-GAN	0.2720	0.0848	0.5712	0.2574
SDNet	0.4096	0.1677	0.7552	0.2955
SwinFusion	0.4164	0.1869	0.7680	0.3183
U2Fusion	0.5378	0.3118	0.9250	0.4068
Ours	0.9937	0.9121	0.9897	0.9758

#### 3.5.2 Phenotypic parameter extraction

**Fig. 7:**
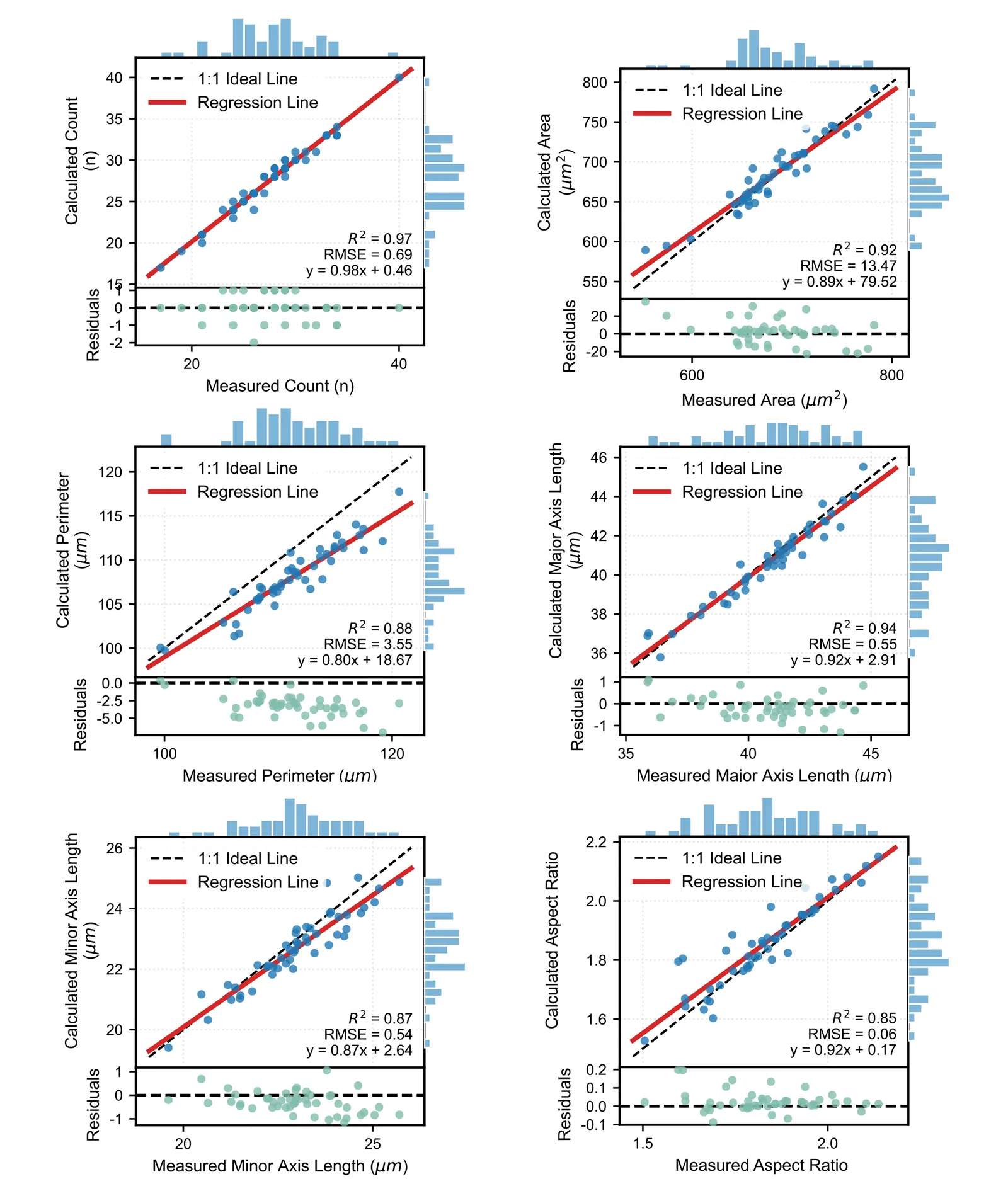
**Evaluation of phenotypic trait extraction accuracy.** Correlation between automatically extracted phenotypic traits and manual measurements, including count, area, perimeter, major axis length, minor axis length, and aspect ratio. The results show strong agreement with the 1:1 reference line, featuring high *R*^2^ values and low RMSE, which demonstrates the robustness and consistency of the automated phenotyping pipeline.

The ultimate goal of stomatal phenotyping is to obtain precise biological and morphological measurements. Based on the instance masks predicted by YOLO, we further computed key geometric traits, including stomatal count, area, perimeter, length, width, and aspect ratio. To quantify the reliability of automated extraction, we compared the computed phenotypic parameters with ground-truth values measured manually under the microscope. At 500× magnification, the calibrated scale was 0.199 μm per pixel. As shown in the scatter plots in Fig. 7, the automatically extracted geometric parameters exhibit very high linear correlation with manual measurements and very low RMSE values. These results indicate that the proposed automated pipeline, namely segmentation driven by unsupervised multi-focus fusion, performs not only well on segmentation metrics but also meets the strict precision requirements of plant phenomics in real agronomic measurements.

## 4 Discussion

### 4.1 Computational efficiency

**Fig. 8:**
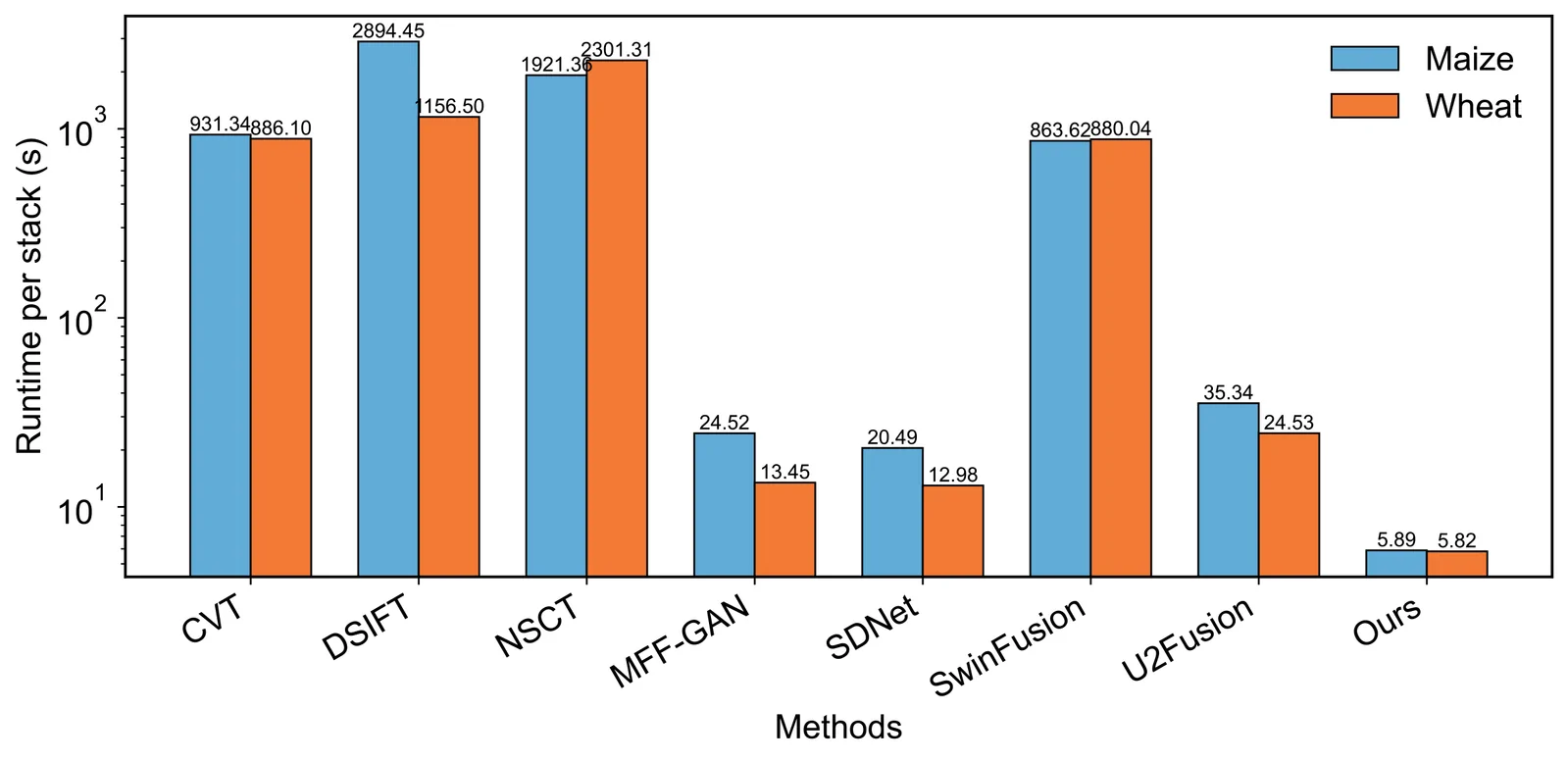
Runtime comparison of different fusion methods on maize and wheat datasets.

To systematically evaluate the efficiency of the proposed fusion model in high-resolution microscopic imaging scenarios, we quantitatively analyzed the running time of different methods on the wheat and maize multi-focus datasets. Because microscopic images in practice are usually high-resolution and involve relatively large focus stacks, the main efficiency metric was the processing time per image stack in seconds. As shown in Fig. 8, under the same hardware conditions, the proposed method shows significant computational advantages on both datasets. For a multi-focus stack containing 21 images, our method requires about 5.8 s on average while still producing high-quality fusion results. Compared with transformer-based advanced methods such as SwinFusion, the proposed method is faster by about two orders of magnitude. Compared with lightweight convolutional fusion methods such as SDNet, it still achieves roughly a fourfold speedup.

It is worth noting that although the wheat and maize datasets differ in image structure, texture complexity, and resolution, the running time of our method varies only slightly across datasets, demonstrating good computational stability. This indicates that the proposed framework is not only effective in fusion quality but also efficient enough for practical deployment in high-throughput microscopic imaging systems.

### 4.2 Generalization across crops

To further assess cross-species and cross-scene generalization of the proposed fusion network, we performed zero-shot testing on rice, soybean, buckwheat, and cotton using a model trained only on the maize dataset. As shown in Fig. S5, the model still generates structurally clear and sharp-edged fusion results on all unseen crops. These results indicate that the model does not overfit crop-specific texture patterns during training, but instead learns general sharpness-related representations from multi-focus image stacks. Therefore, the proposed method can maintain stable fusion performance under different plant species and imaging conditions.

### 4.3 Sensitivity to input order

In multi-focus image acquisition, image stacks are usually collected in order from far to near focal planes, or vice versa, and thus exhibit certain depth continuity. In practice, however, the order of input images may change because of device or human factors.

To test the sensitivity of the model to input order, we designed three input modes on the maize dataset: normal order, reverse order, and random order. The quantitative results in Table 5 show that the three input modes are almost indistinguishable on mainstream fusion metrics such as *Q*_*AB*/*F*_ and *Q*_*CB*_. Only slight fluctuations are observed for VIFF and EN, and the difference between random and reverse order is small. This demonstrates that the model is not sensitive to input order and can maintain stable performance even under unordered or imperfect acquisition conditions. This order invariance mainly arises because the network does not explicitly depend on positional information in the sequence, but instead focuses on local sharpness and multiscale feature modeling.

**Table 5:** Quantitative comparison of multi-focus fusion results under different input conditions.

Dataset	Condition	EN *↑*	MI *↑*	*Q*_AB∕F_*↑*	*Q*_CB_*↑*	VIFF *↑*
Maize	normal	7.43	9.26	0.21	0.41	1.01
	reverse	7.37	8.82	0.21	0.41	0.95
	random	7.42	9.06	0.21	0.41	0.96
Wheat	11 imgs	5.98	5.03	0.27	0.59	0.97
	21 imgs	6.02	6.87	0.22	0.56	1.18
	31 imgs	6.02	9.19	0.20	0.55	1.27

### 4.4 Effect of the number of input images

In practical microscopic imaging, the number of focal-plane images in a stack is often not fixed because acquisition conditions and experimental requirements vary. Therefore, the ability of a model to adapt to different input stack sizes is an important aspect of practicality. To examine this issue, we selected the same region in the wheat dataset and collected stacks with different numbers of images (11, 21, and 31), evaluating ten random positions. As shown in Fig. S6 and Table 5, the model generates visually consistent and clear fusion results under all input sizes. Although some quantitative metrics fluctuate slightly, these differences are almost imperceptible visually.

### 4.5 Future work

The proposed multi-focus fusion method achieves a good balance between efficiency and quality, laying a foundation for practical applications. Future work can focus on system integration and application expansion. In microscopy, the model could be embedded into a self-developed microscope system to build an integrated workflow covering image acquisition, fusion processing, and result output, thereby improving high-throughput imaging efficiency and reducing manual intervention [18]. In agricultural applications, the model could be combined with edge-computing devices and deployed on portable field platforms for rapid acquisition and analysis of crop leaf structures, supporting phenotyping research and precision management [26]. With continued advances in foundation models and unsupervised learning, future studies may also build unsupervised segmentation models for microscopic structures such as stomata, further reducing dependence on manual labels and improving robustness in complex biological imaging scenarios [27].

## 5 Conclusion

This study proposes an unsupervised fusion and phenotyping framework for multi-focus microscopic leaf stomatal images to address the limited depth of field in high-magnification microscopy, where a single image cannot fully capture complete stomatal structures. The method directly models multi-focus image stacks and combines shared 2D feature extraction with 3D cross-focal-plane interaction to fully exploit complementary sharpness information across focal planes, thereby avoiding the error accumulation and information loss caused by conventional iterative fusion. On this basis, we introduce a max-response-guided spatial gating mechanism and dual sharpness priors integrating perceptual features and wavelet high-frequency information, which effectively enhance focused regions and suppress defocus interference, enabling high-quality image reconstruction without all-in-focus ground-truth supervision. Experiments on the maize multi-focus dataset show that the proposed method achieves the best or second-best performance on multiple fusion metrics, including EN (7.43), *Q*_*AB*/*F*_ (0.21), and VIFF (1.01), confirming its advantages in information preservation and structural reconstruction. When the fused results are further applied to a downstream stomatal instance segmentation task, the model achieves mAP50 of 0.9937 and mAP50-95 of 0.9121, and the extracted phenotypic parameters are highly consistent with manual measurements, with stomatal count achieving the highest coefficient of determination *R*^2^ = 0.97. In addition, ablation studies confirm the critical roles of 3D modeling, spatial gating, and dual-prior supervision in the overall framework, while additional experiments demonstrate robust performance under different input sizes and across unseen crop scenarios. Overall, the proposed method provides an effective and scalable solution for multi-focus image fusion in agricultural microscopy and establishes a reliable data foundation for automated stomatal segmentation and high-throughput phenotypic analysis.

## CRediT authorship contribution statement

**Xianglong Shi**: Writing – review & editing, Writing – original draft, Validation, Software, Methodology, Investigation. **Aobo Du**: Writing – review & editing. **Peng Song**: Writing – review & editing, Validation, Resources, Investigation, Conceptualization. **Hui Peng**: Writing – review & editing, Investigation, Conceptualization. **Wanneng Yang**: Writing – review & editing, Validation, Resources, Investigation, Conceptualization. **Ruifang Zhai**: Writing – review & editing, Validation, Resources, Investigation, Conceptualization.

## Data and code availability

Our data and code are available at: https://github.com/Longer-S/UMF-Stomata.

## Declaration of competing interests

The authors declare the following financial interests/personal relationships which may be considered as potential competing interests: Given his roles as Associate Editor, Wanneng Yang had no involvement in the peer review of this article and had no access to information regarding its peer review. Full responsibility for the editorial process for this article was delegated to another journal editor. If there are other authors, they declare that they have no known competing financial interests or personal relationships that could have appeared to influence the work reported in this paper.

## References

[1] Hetherington A.M., Woodward F.I. (2003). The role of stomata in sensing and driving environmental change. Nature.

[2] Xie J., Fernandes S.B., Mayfield-Jones D., Erice G., Choi M., E Lipka A., Leakey A.D.B. (2021). Optical topometry and machine learning to rapidly phenotype stomatal patterning traits for maize QTL mapping. Plant Physiol..

[3] Zhang W., Calla B., Thiruppathi D. (2021). Deep learning-based high-throughput phenotyping accelerates gene discovery for stomatal traits. Plant Physiol..

[4] Millstead L., Jayakody H., Patel H., Kaura V., Petrie P.R., Tomasetig F., Whitty M. (2020). Accelerating automated stomata analysis through simplified sample collection and imaging techniques. Front. Plant Sci..

[5] Xie X., Guo B., Li P., Jiang Q. (2024). OCEANS 2024 - Singapore.

[6] Zhang Y., Gu S., Du J., Huang G., Shi J., Lu X., Wang J., Yang W., Guo X., Zhao C. (2024). Plant microphenotype: from innovative imaging to computational analysis. Plant Biotechnol. J..

[7] Li K., Huang J., Song W., Wang J., Lv S., Wang X. (2019). Automatic segmentation and measurement methods of living stomata of plants based on the CV model. Plant Methods.

[8] Takagi M., Hirata R., Aihara Y., Hayashi Y., Mizutani-Aihara M., Ando E., Yoshimura-Kono M., Tomiyama M., Kinoshita T., Mine A., Toda Y. (2023). Image-based quantification of Arabidopsis thaliana stomatal aperture from leaf images. Plant Cell Physiol..

[9] Li S., Kwok J.T., Wang Y. (2001). Combination of images with diverse focuses using the spatial frequency. Inf. Fusion.

[10] Zhou Z., Li S., Wang B. (2014). Multi-scale weighted gradient-based fusion for multi-focus images. Inf. Fusion.

[11] Aghamaleki J.A., Ghorbani A. (2023). Image fusion using dual tree discrete wavelet transform and weights optimization. Vis. Comput..

[12] Burt P., Adelson E. (1983). The laplacian pyramid as a compact image code. IEEE Trans. Commun..

[13] Ramarao G., Bindu C.H., Murthy T.S.N. (2025). Convolutional laplacian gaussian pyramid approach multimodal medical image fusion. Multimed. Tool. Appl..

[14] Liu Y., Wang L., Cheng J., Li C., Chen X. (2020). Multi-focus image fusion: a Survey of the state of the art. Inf. Fusion.

[15] Luo F., Zhao B., Fuentes J., Zhang X., Ding W., Gu C., Pino L.R. (2025). A review on multi-focus image fusion using deep learning. Neurocomputing.

[16] Li D., Song Z., Quan C., Xu X., Liu C. (2021). Recent advances in image fusion technology in agriculture. Comput. Electron. Agric..

[17] Casado-García A., del-Canto A., Sanz-Saez A., Pérez-López U., Bilbao-Kareaga A., Fritschi F.B., Miranda-Apodaca J., Muñoz-Rueda A., Sillero-Martínez A., Yoldi-Achalandabaso A., Lacuesta M., Heras J. (2020). LabelStoma: a tool for stomata detection based on the YOLO algorithm. Comput. Electron. Agric..

[18] Yao L., von Caemmerer S., Danila F.R. (2025). SCAN: an automated phenotyping tool for real-time capture of leaf stomatal traits in canola. J. Exp. Bot..

[19] Guo L., Dai M., Zhu M. (2012). Multifocus color image fusion based on quaternion curvelet transform. Opt. Express.

[20] Liu Y., Liu S., Wang Z. (2015). Multi-focus image fusion with dense SIFT. Inf. Fusion.

[21] Yang B., Li S., Sun F. (2007). Fourth International Conference on Image and Graphics (ICIG 2007).

[22] Xu H., Ma J., Jiang J., Guo X., Ling H. (2022). U2Fusion: a unified unsupervised image fusion network. IEEE Trans. Pattern Anal. Mach. Intell..

[23] Zhang H., Ma J. (2021). SDNet: a versatile squeeze-and-decomposition network for real-time image fusion. Int. J. Comput. Vis..

[24] Zhang H., Le Z., Shao Z., Xu H., Ma J. (2021). MFF-GAN: an unsupervised generative adversarial network with adaptive and gradient joint constraints for multi-focus image fusion. Inf. Fusion.

[25] Ma J., Tang L., Fan F., Huang J., Mei X., Ma Y. (2022). SwinFusion: cross-domain long-range learning for general image fusion via swin transformer. IEEE/CAA J. Autom. Sin..

[26] Cordier M., Rasti P., Torres C., Rousseau D. (2024). Affordable phenotyping at the edge for high-throughput detection of hypersensitive reaction involving cotyledon loss. Plant Phenomics.

[27] Ravi N., Gabeur V., Hu Y.T., Hu R., Ryali C., Ma T., Khedr H., Rädle R., Rolland C., Gustafson L., Mintun E., Pan J., Alwala K.V., Carion N., Wu C.Y., Girshick R., Dollár P., Feichtenhofer C. (2024). SAM 2: segment anything in images and videos. arXiv:2408.00714.

