# Supplementary material for: UMF-stomata: An unsupervised multi-focus fusion framework for microscopic stomatal phenotyping
Source: Plant Phenomics. 2026 Aug 15;8(4):100266. doi: 10.1016/j.plaphe.2026.100266 (PMC13521141; doi:10.1016/j.plaphe.2026.100266)
Supplement: Supplementary file 1 [file mmc1.docx]

Supplementary Materials

**Supplementary figures**

**Fig. S1.** Max-response-guided spatial gating module.

**Fig. S2.** Residual maps between the fused images and the reference source images from a selected focus stack.

**Fig. S3.** Visualization of multi-focus image stacks and the corresponding decision maps.

**Fig. S4.** Visual comparison of downstream instance segmentation performance using images fused by different methods.

**Fig. S5.** Zero-shot fusion results of the proposed method on unseen crop species, including rice, cotton, soybean, and buckwheat.

**Fig. S6.** Qualitative comparison of fusion results under different numbers of input images on the wheat dataset. “Stacks” denotes the number of images in each input stack.

**Supplementary table**

**Table S1.** Settings of unsupervised deep-learning comparison methods.

| Method | Preprocessing | Stack inference | Training / optimization settings |
| --- | --- | --- | --- |
| MFF-GAN | grayscale/Y;  normalized to [-1,1] | pairwise iterative fusion | Adam, lr 1e-4, batch 32, patch 60, stride 10, 20 epochs |
| SDNet | grayscale/Y;  normalized to [-1,1] | pairwise iterative fusion | Adam, lr 1e-4, batch 32, patch 60, stride 6, 20 epochs |
| U2Fusion | grayscale/Y;  normalized to [0,1] | pairwise iterative fusion | RMSProp, lr 1e-4, batch 18, patch 64 |
| SwinFusion | grayscale/Y;  normalized to [0,1] | pairwise iterative fusion;  window 8, tile 1024, overlap 32 | Adam, lr 2e-4, batch 9, crop 128, EMA 0.999 |
